# Alzheimer's Associated β-Amyloid Protein Inhibits Influenza A Virus and Modulates Viral Interactions with Phagocytes

**DOI:** 10.1371/journal.pone.0101364

**Published:** 2014-07-02

**Authors:** Mitchell R. White, Ruth Kandel, Shweta Tripathi, David Condon, Li Qi, Jeffrey Taubenberger, Kevan L. Hartshorn

**Affiliations:** 1 Department of Medicine, Boston University School of Medicine, Boston, Massachusetts, United States of America; 2 Hebrew Senior Life, Harvard Medical School, Boston, Massachusetts, United States of America; 3 National Institute of Allergy and Infectious Disease, Bethesda, Maryland, United States of America; The Hospital for Sick Children and The University of Toronto, Canada

## Abstract

Accumulation of β-Amyloid (βA) is a key pathogenetic factor in Alzheimer's disease; however, the normal function of βA is unknown. Recent studies have shown that βA can inhibit growth of bacteria and fungi. In this paper we show that βA also inhibits replication of seasonal and pandemic strains of H3N2 and H1N1 influenza A virus (IAV) in vitro. The 42 amino acid fragment of βA (βA42) had greater activity than the 40 amino acid fragment. Direct incubation of the virus with βA42 was needed to achieve optimal inhibition. Using quantitative PCR assays βA42 was shown to reduce viral uptake by epithelial cells after 45 minutes and to reduce supernatant virus at 24 hours post infection. βA42 caused aggregation of IAV particles as detected by light transmission assays and electron and confocal microscopy. βA42 did not stimulate neutrophil H_2_O_2_ production or extracellular trap formation on its own, but it increased both responses stimulated by IAV. In addition, βA42 increased uptake of IAV by neutrophils. βA42 reduced viral protein synthesis in monocytes and reduced IAV-induced interleukin-6 production by these cells. Hence, we demonstrate for the first time that βA has antiviral activity and modulates viral interactions with phagocytes.

## Introduction

βA accumulation is believed to contribute strongly to the pathogenesis of Alzheimer's disease, although the actual physiological function and reason for accumulation of βA in the brain are not known. βA is a fragment of the larger β amyloid precursor protein (APP) which is a transmembrane protein which can be broken down by various proteases into a variety of fragments, including extracellular and intracellular fragments and the fragments βA42 and βA40 which are composed partly of the extracellular and partly of the transmembrane domain of APP. βA40 is more abundant than βA42, but βA42 is the more amyloidogenic species [1]–[3]. βA has been shown to induce neurotoxicity and clinical trials have been focused on reducing its concentration to treat Alzheimer's disease. The structure of βA resembles that of antimicrobial peptides like protegrin and, like protegrin, it can form membrane channels [4]. Importantly, recent studies have demonstrated antibacterial and antifungal activity of βA peptides [5], [6]. There is evidence that these antibacterial and antifungal activities are mediated by the ability of βA peptides to form membrane pores. This implies that βA may play a role in innate defense against infection.

βA accumulation in the brain has been demonstrated in HIV related dementia and recent findings suggest that this results from HIV-induced impairment in proteolysis of βA [7]. Herpes Simplex Virus (HSV) induced encephalitis is associated with βA accumulation in affected areas of the brain, and HSV infection of cell cultures in vitro results in βA accumulation [8]. Of interest, treatment with antivirals reduced accumulation of βA in HSV-infected cell cultures. These findings suggest that viruses that infect the brain could be triggers for accumulation of βA. The triggers of βA production and a full understanding of which cells produce βA in vivo are not clear. βA was detected in plasma, cerebrospinal fluid and culture medium of mixed brain cell cultures in an early study [9]. If βA does function as an innate defense protein one might expect it to be produced during infectious or inflammatory states (as suggested by the HSV findings).

Antimicrobial peptides also frequently function as “alarmins”, triggering recruitment and activation of immune cells [10]. βA peptides are also pro-inflammatory, triggering activation of glial cells and macrophages, and this is thought to relate to neuronal injury [11]. Activation of glial cells by βA has been found to be mediated by TLR2 [12]. These phagocytic cells may also play a role in clearance of βA peptides through phagocytosis and enzymatic degradation of the protein.

In this paper we use influenza A virus (IAV) as a model system to test for antiviral effects of βA peptides. We also study how the peptides alter interactions of IAV with neutrophils and monocytes. We demonstrate that βA peptides have antiviral and immuno-modulatory effects similar to other anti-microbial peptides (e.g. defensins). These studies should open the way for more studies of the effects of βA peptides on other viruses and phagocytes.

## Materials and Methods

### Ethics statement

Blood collection for isolation of neutrophils and monocytes was done with informed consent as approved by the Institutional Review Board of Boston University School of Medicine. The Institutional Review Board specifically approved this study and also approved the consent form for the study. The blood donors were healthy volunteers and they all signed the written consent form prior to each donation.

### Virus Preparations

Philippines 82/H3N2 (Phil82) strain was kindly provided by Dr. E. Margot Anders (Univ. of Melbourne, Melbourne, Australia) and grown in the chorioallantoic fluid of ten day old chicken eggs and purified on a discontinuous sucrose gradient as previously described [13]. Aichi68 H3N2 was obtained from ATCC and PR-8 was kindly provided by Jon Abramson (Wake Forest University) these strains were prepared in eggs in a similar manner. The viruses were dialyzed against PBS to remove sucrose, aliquoted and stored at −80°C until needed. Post thawing the viral stocks contained ∼5×10^8^ infectious focus forming units/ml. The A/California/2009 H1N1 pandemic strain (Cal09) and the A/New York/2001 H1N1 (NY01) seasonal strain were prepared by reverse genetics as described [14].

### βA Preparations

βA42 and βA40 were obtained from AnaSpec, Fremont, CA. βA42 prepared through an alternative method (Hexafluoro-2-propanol, HFIP) was purchased from Phoenix Pharmaceuticals, Burlingame, CA, USA. LL-37 was obtained from Abigent Inc. WRW4 peptide was obtained from Phoenix Pharmaceuticals, Burlingame, CA.

### Hemagglutination (HA) inhibition assay

HA inhibition was measured by serially diluting βA in round bottom 96 well plates (Serocluster U-Vinyl plates; Costar, Cambridge, MA) using phosphate buffered saline (PBS) as a diluent and human type O red cells as described.

### Fluorescent focus assay of IAV infectivity

MDCK cell monolayers were prepared in 96 well plates and grown to confluency. These layers were then infected with diluted IAV preparations for 45 min. at 37°C in PBS. MDCK cells were tested for presence of IAV infected cells after 7 hrs of virus addition using a monoclonal antibody directed against the influenza A viral nucleoprotein (provided by Dr. Nancy Cox, CDC, Atlanta, GA) as previously described. IAV was pre-incubated for 30 min. at 37°C with various concentrations of βA or control buffer, followed by addition of these viral samples to the MDCK cells. In some assays, human bronchial tracheal epithelial cells (HBTE) cells or small airway epithelial cells were used. These cells were purchased from the American Type Culture Collection (Manassas, VA) and propagated in the undifferentiated state in standard tissue culture flasks. For neutralization experiments the multiplicity of infection (MOI or ratio of virus particles to epithelial cells) was 0.1.

### Lactate dehydrogenase (LDH) assay

The LDH assay was performed on MDCK cells infected with Phil82 IAV and βA42 or treated with βA42 alone. Controls included uninfected cells and cells infected with IAV without any peptide added. The assay was performed according to the manufacturer's instructions (Clontech, CA). In brief the assay includes positive and negative controls and is an ELISA. The percent cytotoxicity is obtained from OD values by the formula: OD490_positive control_ − OD490_negative control_ ÷ OD490_positive control_ × Sample OD490.

### Measurement of viral RNA

RNA for the viral M protein was measured using a quantitative real time PCR as previously described [15]. MDCK cells were infected with IAV virus strains incubated for 30 min at 37°C with or without various doses of βA42. RNA extraction was done at 45 min and 24 hrs post infection using Magmax viral RNA isolation kit (Applied Biosytems, Carlsbad, California) as per manufacturer's instructions. For these experiments we used and MOI of 1 since the concentration used on the neutralization assays were too low to register reliably by the PCR assay after 45 min of infection. Both lysed cells and cell supernatant were used for extraction. Viral RNA was also extracted from different concentrations of virus with known FFC/ml which was used as standard series. RNA was reverse transcribed using TaqMan reverse transcription reagents (Applied Biosytems, Carlsbad, California). The reaction mix and the cycle conditions were as per manufacturer's instructions. For real time PCR, primers specific for IAV M protein (Forward AGA CCA ATC CTG TCA CCT CTGA and Reverse: CTG CAG TCC TCG CTC ACT) were used. The primers and TaqMan-labeled probes with non-fluorescent minor groove binder (MGB) moieties were designed manually using the Primer Express software version 3.0 (Applied Biosystems, Carlsbad, California) and were also synthesized by Applied Biosystems. The assay sequences were examined for specificity by nucleotide BLAST. The experiment was performed in a 7500 Real time PCR system (Applied Biosytems, Carlsbad, California) using volume of 20 µL containing 2 ul of template cDNA, 0.9 uM primer 0.25 uM of 6-FAM dye-labeled TaqMan MGB probe (6-FAM-ATT TGT GTT CAC GCT CAC CGT G-MGB), and 1× TaqMan Universal PCR master mix (Applied Biosytems, Carlsbad, California). Thermal cycling proceeded at 50°C for 2 min, 95°C for 10 min followed by 40 cycles of 95°C for 15 s, 60°C for 1 min and 72°C for 30 s. For calculation of FFC/ml from the Cycle threshold (Ct) values, we first plotted a standard curve using the known Log_10_ values of FFC/ml and corresponding Ct values. The Ct values of samples were converted to log 10 values of FFC/ml (x) using the formula y = mx+c where y is the Ct value, m is the slope and c is the intercept. Slope and intercept were calculated from the standard curve using Micosoft Excel. Log_10_ values of FFC/ml were converted to FFC/ml by doing an anti-log (10∧log_10_ value).

### Measurement of viral aggregation by βA and electron microscopy

Viral aggregation caused by βA was measured by assessing light absorbance at 350 nM by suspensions of IAV. This was done using a Perkin Elmer Lambda 35 UV/Vis spectrophotometer. In addition, viral aggregation was assessed using electron microscopy (EM) as described [16]. In brief, βA42 was incubated with Aichi68 IAV at 37°C for 30 minutes, and a 4 µl sample was placed on each copper grid. After the unbound virus was blotted off, the grid was fixed with 4 µl of 2.5% glutaraldehyde for 5 minutes. Samples were stained with 1% sodium phosphotungstate (pH 7.3, Sigma-Aldrich, St. Louis, MO) for 10 seconds, and excess stain was blotted off. The grids were then air dried and stored in a grid box until examined with a Phillips 300 electron microscope (Mahwah, NJ).

### Confocal microscopy

For these experiments the Aichi68 IAV was labeled with Alexa Fluor 594. Alexa Fluor 594 carboxylic acid, succinimidyl ester labeling kit was purchased from Molecular Probes and labeling was carried out using manufacturer's recommendations with some modifications. In brief, concentrated virus stock was incubated with the Alexa Fluor in sodium bicarbonate buffer (pH 8.3) for one hour at room temperature. The preparation was then dialyzed overnight against PBS at 4°C. After this procedure there was no reduction in viral hemagglutination titer. MDCK cells were pre-incubated with the labeled virus for 45 min., followed by washing and fixation using 1% paraformaldehyde. Prior to this the IAV was either pre-incubated with control buffer or βA for 30 minutes at 37°C in the same manner as in the infectious focus assay. Wheat germ agglutinin (WGA)-Oregon Green 488 (4 µg/ml) and DAPI 350 were used to stain the cell membrane and nucleus respectively. Confocal pictures were taken at Zeiss LSM510 (LSEB) on 100× resolution.

### Human Neutrophil and Monocyte Preparation

Neutrophils from healthy volunteers were isolated to >95% purity by using dextran precipitation, followed by Ficoll-Paque gradient separation for the separation of mononuclear cells (layering above the Ficoll-Paque) and neutrophils (below the Ficoll-Paque). The neutrophils were purified further by hypotonic lysis to eliminate any contaminating erythrocytes, as previously described. Cell viability was determined to be >98% by trypan blue staining. The isolated neutrophils were resuspended at the appropriate concentrations in control buffer (PBS) and used within 2 hours. Peripheral blood mononuclear cells (PBMCs) were taken from the layer above Ficoll-Paque and washed several times in PBS. Monocytes were isolated from the PBMC preparations by negative selection using magnetic beads using a Miltenyi monocyte isolation kit (catalogue number 130-091-153).

### Measurement of IAV uptake by neutrophils and monocytes

Fluorescein isothiocyanate (FITC)-labeled IAV (Phil82 strain) was prepared and uptake of virus by neutrophils or monocytes was measured by flow cytometry as described [17]. In brief, IAV was treated with various doses of βA peptides for 30 min at 37°C. Then it was incubated with cells for 45 minutes at 37°C in presence of control buffer. Trypan blue (0.2 mg/ml) was added to these samples to quench extracellular fluorescence. Following washing, the neutrophils were fixed with 1% paraformaldehyde and neutrophil and monocyte associated fluorescence was measured using flow cytometry. The mean cell fluorescence (>2000 cells counted per sample) was measured. For the experiments involving neutrophils and monocytes (e.g. viral uptake, NET formation, H2O2 production and cytokine generation the MOI was ∼40).

### Assessment of neutrophil extracellular trap (NET) formation

For NETs study, neutrophils were resuspended in PBS supplemented with Ca^2+^ and Mg^2+^ and allowed to adhere on Poly-L-Lysine coated 96 well plates or on glass bottom culture dishes (MatTek corporation, Ashland, MA) for 1 hr in a CO_2_ incubator. After 1 hr, unadhered cells were removed and adhered cells were incubated for 3 hrs in a CO_2_ incubator with Phil82 virus after preincubation of the virus (30 min, 37°C) with βA42. For quantitative assessment of NETs formation, Sytox green (Life Technologies) was added to samples on 96 wells plate after the 3 hr incubation period and the plate was read on POLARstar OPTIMA fluorescent plate reader (BMG Labtech, Durham NC). Immediately after reading the plate, the plate was also photographed on a fluorescent microscope as well.

### Assay of tumor necrosis factor alpha (TNFα) and IL-6 production by human monocytes

Human peripheral blood monocytes were isolated by magnetic bead separation as described above and infected with an MOI of ∼50 of Phil82 IAV for 45 min at 37°C. The virus was either used alone or after 45 min incubation with various concentrations of βA. After this the cells were pelleted, washed with PBS and then cultured for 18 hrs at 37°C in RPMI with 10% autologous serum in a CO_2_ incubator. After 18 hrs, supernatant was collected and assayed for TNFα using a sandwich ELISA method (catalog number MTNFAI; Endogen), following the manufacturer's instructions. The IL-6 ELISA was performed using a kit from Abcam (Cambridge, MA, USA).

### Measurement of neutrophil H_2_O_2_ production

H_2_O_2_ production was measured by assessing reduction in scopoletin fluorescence as previously described. Measurements were made using a POLARstar OPTIMA fluorescent plate reader (BMG Labtech, Durham NC).

### Statistics

Statistical comparisons were made using Student's paired, two-tailed *t* test or ANOVA with post hoc test (Tukey's). ANOVA was used for multiple comparisons to a single control.

## Results

### Neutralization of IAV strains by βA preparations

**Figure 1:**
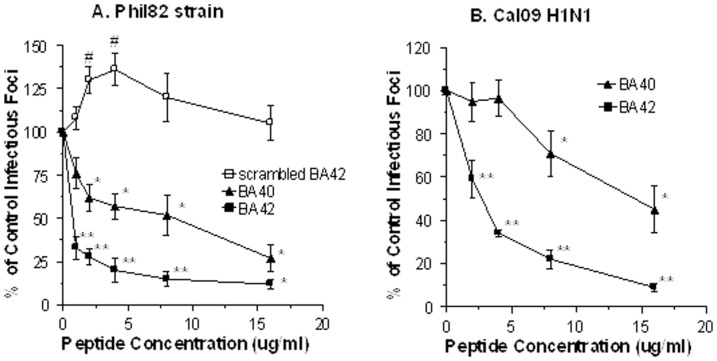
Viral neutralizing activity of βA42 and 40 for seasonal H3N2 and pandemic H1N1 strains of IAV. Aliquots of the Phil82 H3N2 (panel A) or Cal09 H1N1 (panel B) viral strains were incubated with the indicated concentrations of βA42 or 40 or a scrambled version of βA42 and then these samples were used to infect HBTE cell monolayers and tested for infectious foci 7 hrs later using anti-nucleoprotein antibodies and fluorescence detection. * indicates significant reduction in infectivity compared to control (p<0.05; n = 4 experiments) ** indicates p<0.02 compared with βA40, scrambled βA42 and control (ANOVA analysis) # indicates increased infectivity compared to control.

As shown in figure 1A, βA42 and βA40 peptides significantly inhibited infectivity of a seasonal H3N2 strain of IAV (Phil82) to infect HTBE cells. For neutralization experiments we used an MOI of 0.1 to enable counting of infected cells. A scrambled version of βA42 did not have any activity in this assay. βA42 had significantly greater anti-viral activity than βA40. As shown in figure 1 panel B, βA40 and 42 also inhibited an H1N1 pandemic strain from 2009 (Cal09). Again the effect of βA42 was significantly greater than that of βA40. BA42 also reduced infectivity of IAV for small airway epithelial cells (e.g., 16 µg/ml of BA42 reduced infectivity of Phil82 to 29±12% of control and Cal09 to 47±15% of control; n = 6; p<0.01 compared with viral control in both cases; data not shown).

**Figure 2:**
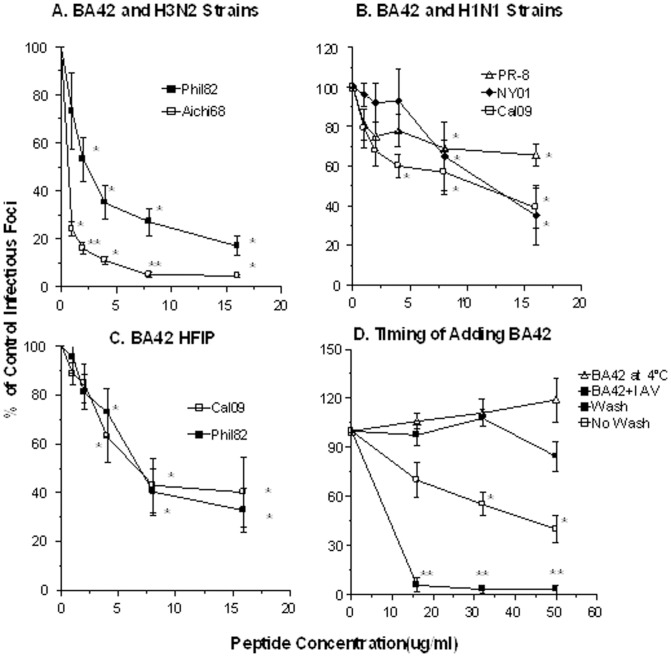
Viral neutralizing activity of βA42 preparations for various IAV strains. Experiments were carried out as in figure 1 except that MDCK cells were used instead of HTBE cells. Panel A shows inhibition of two H3N2 strains as indicated. Panel B shows inhibition of three H1N1 strains. Panel C shows effects of a different commercially available preparation of βA42 (called βA42 HFIP) against Phil82 and Cal09 strains. Panel D shows the effect of βA42 on infection of A549 cells by Aichi68 IAV and compares the effects of pre-incubating the virus with βA42 as in panel A (“pre-incubate with IAV”) with pre-incubating the cells with βA42. In the latter case, the βA42 was either left in the cell media when virus was added (“no wash”) or washed off prior to adding the virus (“wash”). Although in the “no wash” setting infectivity was significantly reduced this effect was significantly less than when the virus was pre-incubated with βA42 (p<0.01). Finally panel D also shows the effect of pre-incubating cells with IAV at 4°C to allow binding, followed by adding βA42, and increasing the temperature to 37°C (“βA42 at 4°C”). * indicates significant reduction in viral infectivity compared with control (virus alone) (p<0.01). ** indicates where the Aichi68 strain was inhibited significantly more than the Phil82 strain (panel A). Results shown are mean±SEM of 4 or more experiments.

We used MDCK cells to make additional comparisons of the effects of BA42 on infectivity of several different H3N2 and H1N1 strains (figure 2A and B). Of interest, βA42 had significantly greater neutralizing activity against the Aichi68 pandemic H3N2 strain than against the seasonal Phil82 H3N2 strain (figure 2A). The activity of βA42 against Cal09 was equal to or greater than its activity against the seasonal NY01 H1N1 strain or the mouse adapted PR-8 H1N1 strain. Note, however, that the antiviral activity of βA42 for Cal09 was somewhat less in MDCK cells (figure 2C) than in HTBE cells (figure 1). We also tested the activity of an additional preparation of βA42 against Phil82 and Cal09 strains. This βA42 protein was prepared using HFIP to avoid protein self-aggregation during purification. As shown in figure 2C, the βA42 HFIP preparation inhibited both IAV strains.

The antiviral effect of βA42 was also seen with A549 cells as shown with the Aichi68 H3N2 virus (figure 2D; “Virus+βA42”). In all experiments described thus far the viruses were pre-incubated with βA42 prior to adding the mixture to cells. In figure 2D we also tested the effects of adding βA42 to cells prior to adding the virus. The βA42 was either left in the medium when virus was added (“no wash”) or washed off prior to adding the virus (“wash”). As shown in figure 2D, washing off the βA42 completely eliminated the antiviral effect, while leaving βA42 in the medium resulted in a partial inhibition much reduced as compared to the experiments in which virus and βA42 were pre-incubated together. This suggests that the antiviral effect of βA42 is largely mediated by its direct interaction with the virus. We also tested incubation of the cells with virus for 45 at 4°C followed by adding βA42 and then raising the temperature to 37°C (“BA42 at 4°C”). Using this method no evident inhibition of infection was observed. This suggested also that the interaction of βA42 with free virus (prior to viral binding to cells) was critical.

To be sure that the observed antiviral activity of βA42 was not an artifact of cell injury caused by the peptide we performed LDH assays on MDCK cells treated with βA42 alone (at 20 and 40 µg/ml) or βA42 at the same concentrations in the presence of Phil82 IAV. The conditions for these experiments were the same as for the infectious focus assays. The assay uses a positive and negative control to determine the % of cellular cytotoxicity. βA42 alone or in presence of virus did not increase cytotoxicity significantly over untreated MDCK cells and all treated cells were ≤1% cytotoxicity (data not shown; 4 separate experiments done).

**Figure 3:**
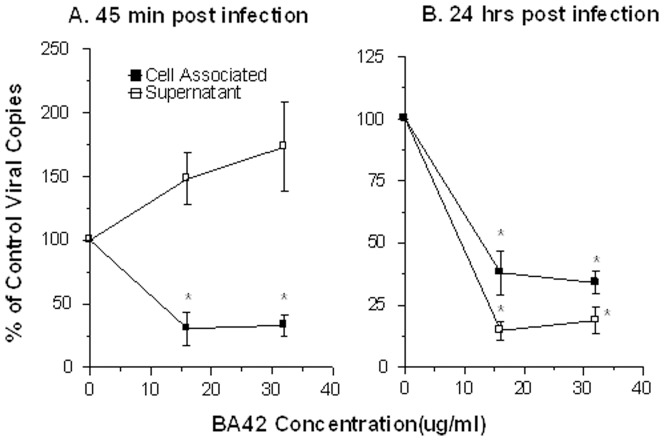
Effects of βA42 on viral internalization and viral replication in MDCK cells as determined by quantitative RT-PCR. In panels A, Phil82 IAV was pre-incubated with the indicated concentrations of βA42, followed by incubation of these samples for 45 min with MDCK cells as in figure 1. The cell supernatant and cell pellets were then harvested and viral RNA was extracted followed by PCR assay to determine the amount of virus present in cells and supernatant. βA42 significantly reduced the amount of virus taken up by cells after 45 min of infection. There was a trend to increased virus in the supernatant after 45 min of infection but this was not significant. Panel B shows the results of cell and supernatant assays after 24 hrs of infection. βA42 significantly reduced the amount of virus in both cell and supernatant at this time. * indicates p<0.05 compared with control. Results are mean±SEM of three experiments.

To further probe the mechanism of antiviral activity of βA42 we performed quantitative PCR assays for the viral M protein to determine if the peptide reduced viral uptake by epithelial cells at 45 min after infection. For these experiments we used and MOI of 1 since the concentration used on the neutralization assays were too low to register reliably by the PCR assay after 45 min of infection. As shown in figure 3A, βA42 did significantly reduce the amount of viral RNA taken up into the MDCK cells while increasing the amount of virus remaining in the supernatant. This finding indicates that the mechanism of antiviral activity of βA42 involves at least in part reduced uptake by epithelial cells. We also tested whether viral RNA present in cells and cell supernatants was reduced by βA42 at 20 hrs after infection. Figure 3B shows a strong reduction in cellular or supernatant viral RNA at this time in samples where the virus was pre-treated by βA42.

We have previously reported that HNPs, retrocyclins and LL-37 do not inhibit viral hemagglutination activity, despite the fact that all these peptides cause viral neutralization. Similarly βA peptides did not inhibit HA activity of the Phil82 strain (data not shown).

### Viral aggregation in response to βA peptides

**Figure 4:**
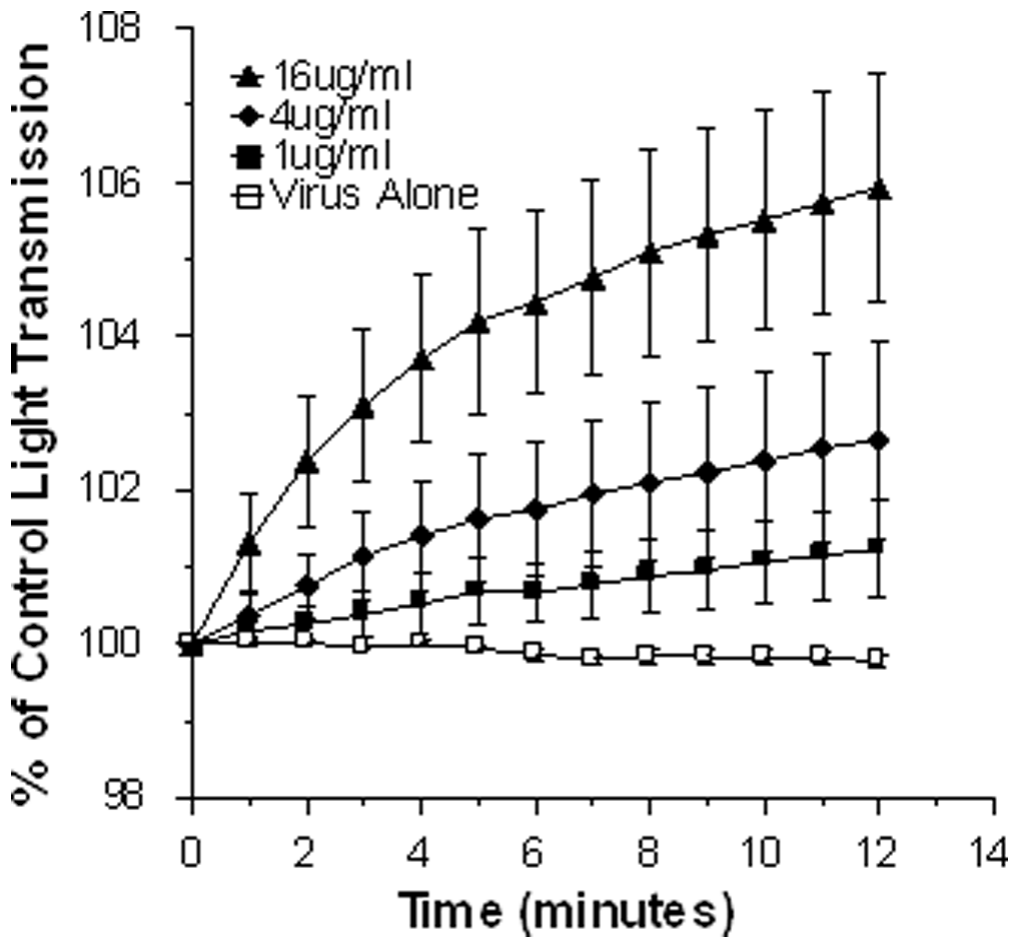
Viral aggregation induced by βA42. Aggregation of the Aichi68 H3N2 viral strain was assessed based on increased light absorbance at 350(n = 5; p<0.02) at the 16 µg/ml concentration of βA42 as compared to light transmission through the control virus preparation.

**Figure 5:**
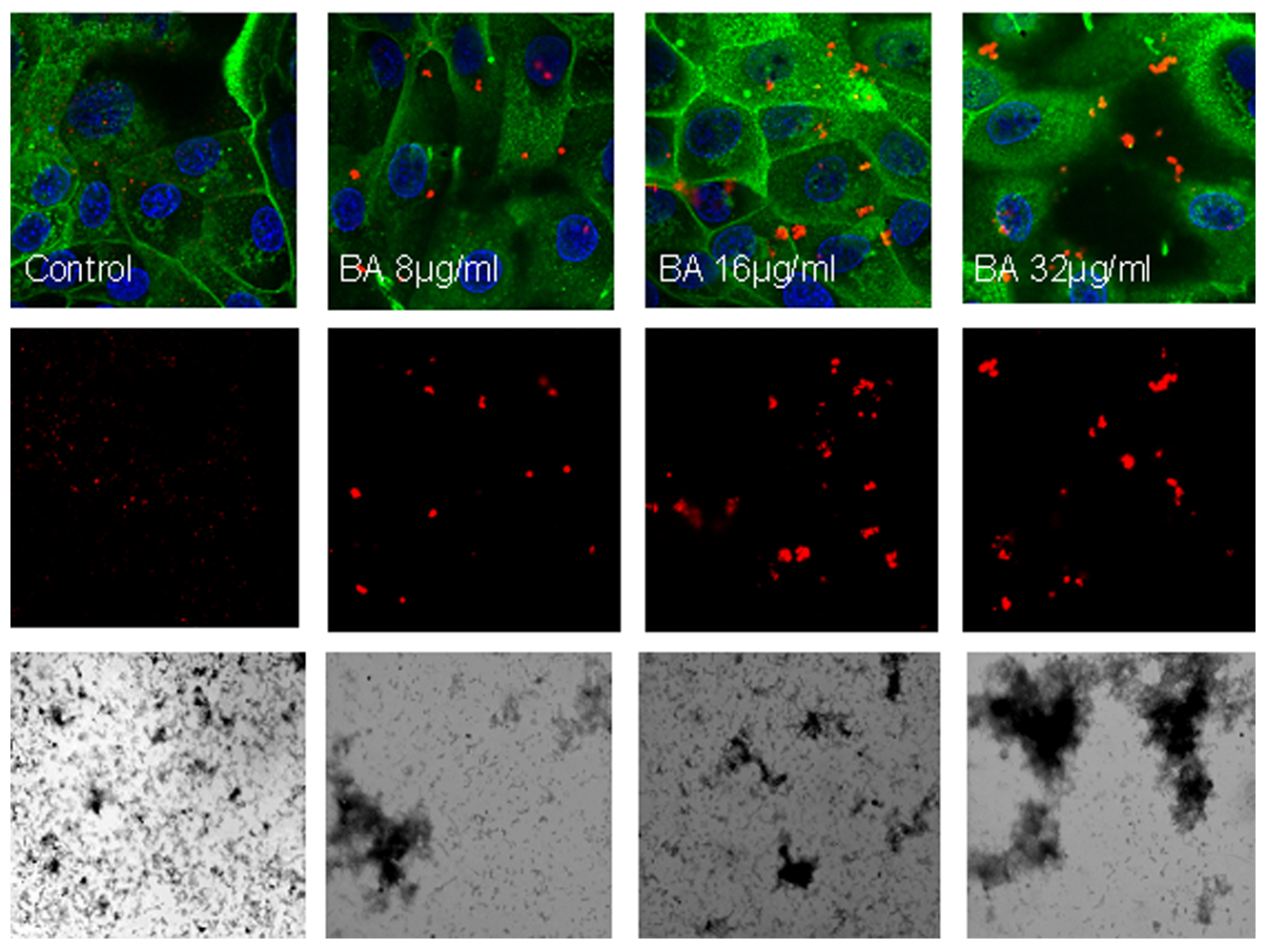
Confocal and electron microscopy demonstrating viral aggregates formed by βA42. Alexafluor 594-labeled Aichi68 IAV was incubated with control buffer or βA42 at the indicated concentrations. After this the virus +/- βA42 samples were added to MDCK cell monolayers as described in Methods. The virus appears red, cell nuclei were stained with Dapi 350 and appear blue, and cell membranes were labeled with WGA-Oregon Green 488. The upper pictures show the same fields as those below but the middle pictures only show the wavelength of virus. Aggregates were also observed on electron microscopy (bottom row of pictures). Results are representative of three similar experiments. The pictures were taken at 100× magnification.

Some other innate viral inhibitors like human neutrophil defensins (HNPs), collectins and ficolins induce viral aggregation. As shown in figure 4, βA42 caused significant dose related aggregation of H3N2 IAV (Aichi68 strain) as assessed by light transmission assay. Figure 5 demonstrates the aggregates formed by βA42 using confocal microscopy. In the control panel the virus (red color) is barely visible. In the βA42 treated samples definite visible aggregates are present. The aggregates were also demonstrated using electron microscopy (bottom panels figure 5).

### Effects of βA peptides on neutrophil interactions with IAV

**Figure 6:**
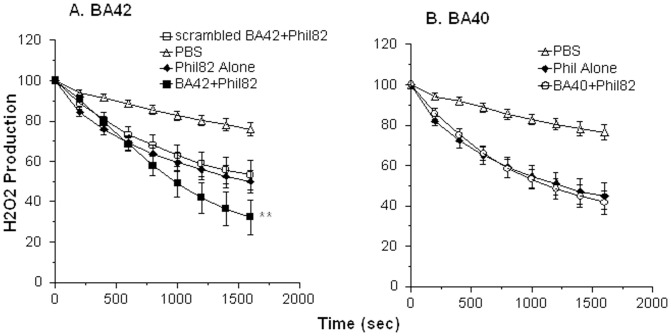
Neutrophil H_2_O_2_ responses to IAV alone or IAV treated with βA42 or βA40. Human neutrophils were treated with buffer alone (PBS), IAV alone (Phil82) or IAV that had been pre-treated with βA42 or scrambled βA42 (panel A) or βA40 (panel B) (all at 16 µg/ml). βA42 caused a significant increase in H_2_O_2_ response compared to IAV alone or IAV pre-treated with the scrambled peptide of βA40 (indicated by **). Note that the latter two proteins did not increase the response compared to IAV alone. Results are mean±SEM from 5 experiments (each with a separate neutrophil donor).

**Figure 7:**
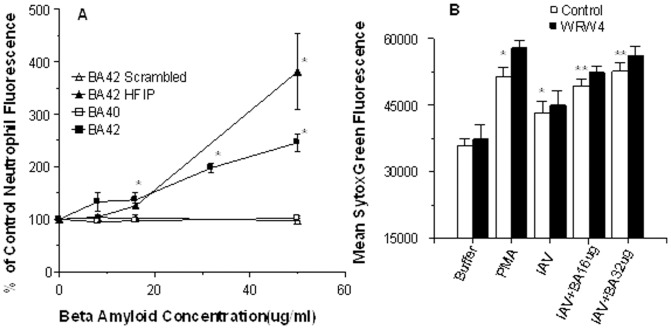
βA42 increases neutrophil uptake of IAV and IAV-induced NET formation. In panel A, neutrophils were treated with FITC-labeled Phil82 IAV alone or virus pre-incubated with the indicated peptides. βA42 and βA42 HFIP significantly increased viral uptake by the neutrophils (indicated by * for both; n = 4; p<0.03 compared with control). The amounts of βA shown are those that were initially incubated with IAV prior to a dilution which occurred when these samples were added to the neutrophils. For example where 50 µg/ml is shown in figure 7A or 9B the final concentration with cells was 14 µg/ml. The scrambled peptide and βA40 did not increase viral uptake. Panel B shows NET formation in response to control buffer, IAV alone, βA42 alone or IAV pre-incubated with either 16 or 32 µg/ml of βA42. Sytox green fluorescence was measured by plate reading fluorometer after 30 min of treatment at 37°C. The results shown are mean±SEM of 5 experiments using separate neutrophil donors. The open bars are results of the stimuli in control cells. The black bars are results obtained with cells pre-treated with the WRW4 peptide which inhibits responses mediated by formyl peptide receptors. * indicates p<0.01 vs control buffer. ** indicates p<0.01 vs IAV alone. The WRW4 peptide did not reduce responses to any stimulus.

**Figure 8:**
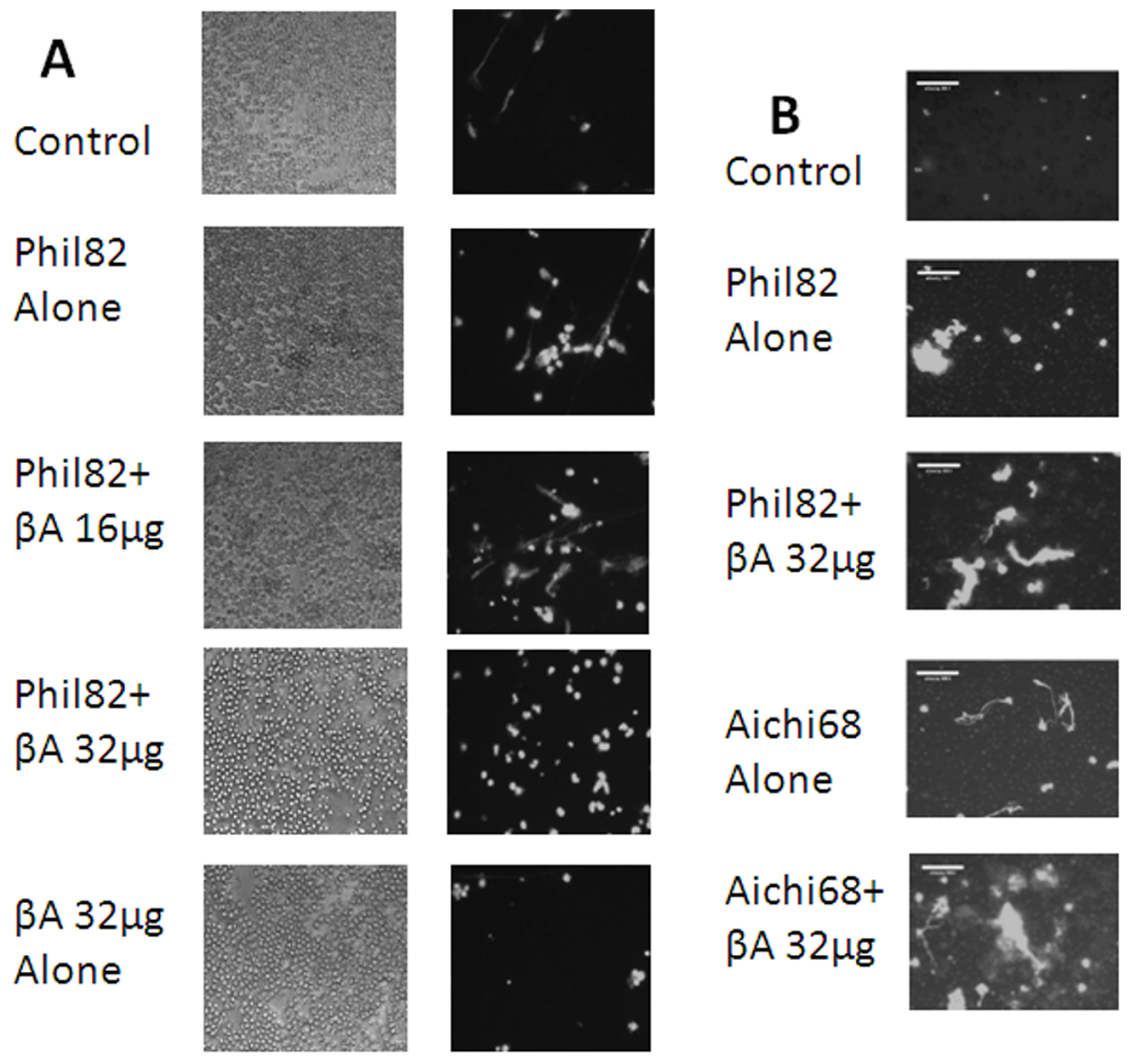
Fluorescent micrographs of NET formation in response to IAV and/or βA42. Panel A: Results are representative of 4 experiments using different neutrophil donors. The figures to the left show Sytox green fluorescence and those to the right in this panel show the same fields under phase contrast microscopy to illustrate how many neutrophils were present. These pictures were taken at 40× and enlarged to match the size of the cells in panel B. Panel B are representative of another set of 3 experiments using Phil82 or Aichi68 viral strains as indicated phase and show Sytox green fluorescence taken at 100× magnification.

For neutrophil and monocyte experiments we used considerably higher concentrations of IAV (i.e. MOI of ∼40) at which the virus acts as an agonist for phagocytes as previously described [18]. Our goal was to determine if βA peptides modulate this agonist activity. IAV alone triggers a respiratory burst response in human neutrophils and this response is increased by pre-incubating IAV with collectins. We tested the various preparations of βA alone and these did not provoke any neutrophil H_2_O_2_ response (data not shown). Pre-incubation of Phil82 IAV with βA42 resulted in a significant increase in the IAV-induced response (figure 6A). βA42 HFIP caused a similar significant increase in H_2_O_2_ production (data not shown). In contrast, scrambled βA42 (panel A) and βA40 (panel B) did not significant increase H_2_O_2_ production beyond that induced by IAV alone. Pre-incubation of IAV with βA42 resulted in a significant increase in neutrophil uptake of IAV (figure 7A). Pre-incubation of IAV with βA42 also increased production of NETs in response to virus (figure 7B). βA42 has been shown to bind to formyl peptide receptors on glial cells leading to cellular responses [19]. In view of this we tested the effects of the formyl peptide receptor blocker on NET formation. This blocker, WRW4, had no effect on NET formation in response to IAV or IAV+βA42 (figure 7B). We tested two formyl peptide receptor agonist, fMLP and WKYVM and neither of these induced NET formation (data not shown). Figure 8 shows fluorescent micrographs indicating increased IAV-induced NET formation when either Phil82 or Aichi68 strains of IAV were pre-incubated with βA42. βA42 alone did not induce any increase in NET response compared to buffer alone.

### Effects of βA on monocyte interactions with IAV

**Figure 9:**
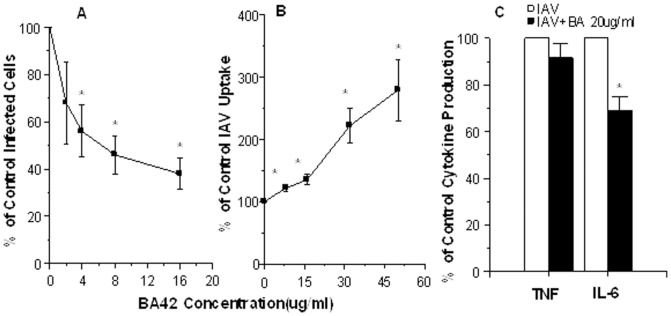
Effects of βA42 on interactions of IAV with monocytes: viral protein synthesis (panel A), viral uptake (panel B) and virus-induced cytokine generation (panel C). Adherent peripheral blood monocytes were infected with Phil82 IAV alone or Phil82 IAV treated with the indicated doses of βA42. In panel A the presence of viral nucleoprotein in the cells was tested as in figure 1 after 24 hours of incubation. * indicates significant reduction compared to virus alone (p<0.03; n = 4 separate blood donors). In panel B the ability of monocytes to take up fluorescently labeled IAV after a 45 min incubation was tested. Increased uptake (p<0.05; n = 4) is indicated by *. In panel C the monocytes were incubated with IAV+/−peptide for 24 hrs and then TNF or IL-6 release into the supernatant was measured by ELISA. IAV alone stimulated increased cytokine production by the cells (see Results section). IL-6 production was significantly reduced by pre-incubation of the virus with βA42 (p<0.02; n = 4; indicated by *). TNF production was not reduced by βA42 (n = 8).

βA42 significantly reduced the percentage of monocytes expressing viral nucleoprotein at 18 hrs after infection (figure 9A). Of interest, pre-incubation with βA42 resulted in increased uptake of fluorescently labeled IAV by monocytes after a 45 min incubation (figure 9B). Phil82 IAV induced robust TNF and IL-6 production after 18 hrs of infection as previously reported. The mean TNF production from uninfected control, IAV-infected cells and cells exposed to 20 µg/ml of βA42 alone were: 6±2, 52±14, and 4±2 ng/ml, respectively (p<0.001 for IAV treated vs control; n = 8). Similarly, IL-6 levels for control, IAV infected or βA42 treated cells were: 16±3, 36±6, and 20±4 ng/ml (p<0.01 for IAV-infected vs control; n = 4). The monocytes were treated with βA42 alone did not make significantly more TNF or IL-6 than controls in these experiments. Pre-incubation of the virus with βA42 did not alter IAV-induced TNF production but did significantly reduce the IL-6 response (figure 9C; results expressed as % of viral control).

## Discussion

We demonstrate for the first time that βA peptides have antiviral activity. The βA peptides had antiviral activity not only in MDCK cells but in more physiological cell models (primary tracheobronchial or small airway cells). The antiviral activity of βA42 appeared to be somewhat greater in the HTBE cells than in MDCK cells. The antiviral activity of βA42 was significantly greater than that of βA40. This is in agreement with findings of Soscia et al that βA42 has greater anti-bacterial and anti-fungal activity than βA40 [6]. It is notable that the peptides also inhibited the Cal09 H1N1 pandemic strain which is not inhibited by several other innate inhibitors including the collectins, MBL and SP-D, or pentraxin [16], [20]. Using quantitative PCR assays we show that βA42 reduces uptake of IAV by epithelial cells which accounts at least in part for its antiviral activity. We also show that βA42 reduces both cellular and supernatant viral RNA at 24 hours after infection. In addition, we show that it is critical to pre-incubate the virus with βA42 to achieve optimal inhibition. Hence, the peptide appears to mainly act on the virus itself rather than on the cell to achieve neutralization. In agreement with prior findings obtained with defensins, retrocyclins and LL-37, βA peptides did not inhibit HA activity of IAV [15], [21]. Our results further suggest that the ability of βA42 to cause viral aggregation may in part account for viral inhibition. Specifically, pre-incubation of epithelial cells with the virus at 4C (allowing binding of virus but not internalization), followed by addition of βA42 caused no inhibition of infectivity.

The findings that βA42 is able to induce viral aggregation is of interest and may reflect the self-aggregating potential of this peptide [1]. We have proposed a similar mechanism for the ability of HNPs and retrocyclins to induce viral aggregation [21], [22]. In contrast, LL-37 does not induce any viral aggregation and inhibits infectivity at a step after viral internalization in the cell [15]. The mechanism of antiviral activity of βA peptides involves reduced viral uptake by epithelial cells, possibly as a result of viral aggregation. We cannot exclude the possibility that βA peptides also alter viral membranes to reduce infectivity. Future studies with more sensitive EM techniques might clarify this.

Antimicrobial peptides have many immune modulating effects and recent studies suggest that βA peptides have similar effects. We show that βA42 increases neutrophil uptake of IAV and also increases neutrophil respiratory burst responses to the virus. Other types of amyloid fibrils have been shown to induce NET formation [23] and we now show that βA42 induces NETs but only in the presence of virus. Since βA42 has been reported to mediate some of its effects on glial cells through formyl peptide receptors [12] which are also present on neutrophils [24], we tested whether a blocker of these receptors would alter the enhanced NET formation caused by βA42. This blocker had no effect in this assay so it does not appear that this effect of βA42 is mediated by formyl peptide receptors. Of interest, βA42 significantly increased monocyte uptake of IAV after 45 min of incubation but viral protein production was reduced at 20 hrs. This indicates that the virus taken up in the presence of βA42 does not replicate in the cells. Based on these findings βA42 appears to help in viral clearance by phagocytes. βA42 reduced IAV-induced IL-6 but not TNF production by these cells. Further studies of the immuno-modulatory effects of βA peptides, or their interaction with other innate immune molecules, will be of great interest [25].

βA peptides have been found at low concentration in human serum and at high concentration in brain tissue of patients with Alzheimer's disease. It is not clear if local production of βA peptides occurs during inflammation in other parts of the body. Since IAV predominantly replicates in the respiratory tract significant amounts of βA would need to be present in respiratory lining fluids or lung to participate in viral inhibition. Avian strains (e.g. H5N1) can cause direct brain infection in mice although CNS infection during human influenza is extremely rare [26]. In addition, neurological sequelae of IAV infection are well documented (e.g. the encephalitis which resulted from the 1918 pandemic). It has not been determined if such sequelae are associated with βA accumulation in the brain. In any case, our findings may have more relevance to other viruses that infect the CNS including HIV and HSV. HIV related dementia is associated with accumulation of βA in the brain [7]. Strong associations of HSV infection with βA production and accumulation have been reported as well [27]. Cytomegalovirus infection has also been associated with Alzheimer's disease [28]. Further studies of antiviral activity of βA against these viruses would be of great interest. It would also be of great interest to determine if IAV induces increased βA production in various cell types. Our studies show that βA has both antiviral activity and strong immuno-modulatory effects using IAV as a model system and extension of these studies to other viruses, including those linked to Alzheimer's disease or brain infection will be of great interest.
